# Systematic evaluation of parameters in RNA bisulfite sequencing data generation and analysis

**DOI:** 10.1093/nargab/lqac045

**Published:** 2022-06-03

**Authors:** Zachary Johnson, Xiguang Xu, Christina Pacholec, Hehuang Xie

**Affiliations:** Epigenomics and Computational Biology Lab, Fralin Life Sciences Institute, Virginia Tech, Blacksburg, VA 24061, USA; Genetics, Bioinformatics and Computational Biology Program, Virginia Tech, Blacksburg, VA 24061, USA; Epigenomics and Computational Biology Lab, Fralin Life Sciences Institute, Virginia Tech, Blacksburg, VA 24061, USA; Department of Biomedical Sciences and Pathobiology, Virginia-Maryland College of Veterinary Medicine; Virginia Tech, Blacksburg, VA 24061, USA; Epigenomics and Computational Biology Lab, Fralin Life Sciences Institute, Virginia Tech, Blacksburg, VA 24061, USA; Department of Biomedical Sciences and Pathobiology, Virginia-Maryland College of Veterinary Medicine; Virginia Tech, Blacksburg, VA 24061, USA; Epigenomics and Computational Biology Lab, Fralin Life Sciences Institute, Virginia Tech, Blacksburg, VA 24061, USA; Genetics, Bioinformatics and Computational Biology Program, Virginia Tech, Blacksburg, VA 24061, USA; Department of Biomedical Sciences and Pathobiology, Virginia-Maryland College of Veterinary Medicine; Virginia Tech, Blacksburg, VA 24061, USA; Translational Biology, Medicine and Health Program, Virginia Tech, Blacksburg, VA 24061, USA; School of Neuroscience, Virginia Tech, Blacksburg, VA 24061, USA

## Abstract

The presence of 5-methylcytosine (m^5^C) in RNA molecules has been known for decades and its importance in regulating RNA metabolism has gradually become appreciated. Despite recent advances made in the functional and mechanistic understanding of RNA m^5^C modifications, the detection and quantification of methylated RNA remains a challenge. In this study, we compared four library construction procedures for RNA bisulfite sequencing and implemented an analytical pipeline to assess the key parameters in the process of m^5^C calling. We found that RNA fragmentation after bisulfite conversion increased the yield significantly, and an additional high temperature treatment improved bisulfite conversion efficiency especially for sequence reads mapped to the mitochondrial transcriptome. Using Unique Molecular Identifiers (UMIs), we observed that PCR favors the amplification of unmethylated templates. The low sequencing quality of bisulfite-converted bases is a major contributor to the methylation artifacts. In addition, we found that mitochondrial transcripts are frequently resistant to bisulfite conversion and no p-m5C sites with high confidence could be identified on mitochondrial mRNAs. Taken together, this study reveals the various sources of artifacts in RNA bisulfite sequencing data and provides an improved experimental procedure together with analytical methodology.

## INTRODUCTION

Post-transcriptional modification of RNA molecules plays a fundamental role in the regulation of RNA function and metabolism (1–4). Among the more than 170 types of RNA modifications that have been identified (5), RNA 5-methylcytosine (m^5^C) is one of the most well-known and widely present in transfer RNAs (tRNAs), ribosomal RNAs (rRNAs) and messenger RNAs (mRNAs) (6–8). RNA m^5^C modification in tRNAs, mediated by DNA methyltransferase 2 (DMNT2) and members of the NOP2/Sun RNA methyltransferase enzyme family (NSUN) (6,9–11), promote tRNA stability and protein synthesis (9)). RNA m^5^C modification in rRNAs, introduced by NSUN5, serves as a conserved mechanism in rRNA-mediated translational regulation (12). Compared to tRNAs and rRNAs, mRNAs carry relatively few m^5^C modifications, the functions of which have been better understood in recent years (8,13–16). Specifically, the m^5^C modification promotes the export of mRNAs from the nucleus to the cytoplasm *via* the RNA binding protein ALYREF (16), stabilizes mRNAs by facilitating the binding of the m^5^C reader protein YBX1 (17–19), and modulates mRNA translation efficiency (20). Moreover, mRNA m^5^C modification is involved in diverse physiological and pathological conditions including facilitating the maternal-to-zygotic transition in early embryos of zebrafish (18), promoting ovarian germ line stem cell development in drosophila (19), and driving the pathogenesis of bladder cancer in humans (17).

Along with advances in high-throughput sequencing, RNA bisulfite sequencing (RNA BS-seq) was developed and widely used for the identification of RNA m^5^C modification at single nucleotide resolution (8,14,16,21,22). Despite the successful confirmation of m^5^C sites in tRNAs (9,23,24) and rRNAs (7,12) using RNA BS-seq, it remains a challenge to obtain reproducible sets of m^5^C in mRNAs, even among biological replicates. Currently, a wide range of m^5^C sites in the mammalian transcriptome has been reported, ranging from <100 to >10 000 sites per transcriptome (8,14–16,22). Such a large variation in the number of m^5^C sites determined in mRNAs is speculated to be associated with differences in experimental versus computational approaches including inefficient bisulfite conversion, sequencing data-quality controls, methylation calling, and methylation filtering strategies (Supplementary Table S1) (14–16,22,25,26). From an experimental aspect, several versions of RNA BS-seq library construction protocols have been published (14–16,22). The primary differences in these protocols lie in the timing of RNA fragmentation, the temperature and duration of thermal conditions during bisulfite conversion, and the usage of ACT or regular random hexamers for first strand cDNA synthesis. Despite an elegant toolkit meRanTk (25) implemented to provide accurate sequence mapping, methylation calling, and high-confidence filtering; the pipelines used to process RNA BS-seq data vary across different research groups.

Recent studies utilizing high-stringency bisulfite conditions, a ‘C-cutoff’ of RNA BS-seq reads, and other statistical techniques have identified hundreds of high-confidence m^5^C sites in mRNAs in mouse and human tissues (20,22,27). mRNAs carrying high-confidence m^5^C sites were found to be enriched in the mitochondrial gene pathway (22). Research groups focusing on non-coding RNAs have identified the m^5^C modification of mitochondrial tRNAs and one rRNA (11,28–30), indicating the presence of NSUN2 (29,31), NSUN3 (32,33) and NSUN4 (30,34–36) activity within the mitochondrial complex. Some studies identified methylated mRNAs originating from the mitochondrial genome (14,16,22), however, other studies were unable to support this finding (15,37).

Despite the promising results obtained in recent RNA BS-seq studies, it remains a challenge to select an ideal experimental protocol for library construction and appropriate parameters in the data processing procedure to accurately identify m^5^C sites. In this study, we compared four different protocols for RNA BS-seq library construction. RNA samples isolated from the mitochondria of mouse neural stem cells (NSCs) was used as starting materials. The small size of mitochondrial transcriptome helps in producing sequences with sufficient read depth and minimizing artifacts resulted from multi-mapping, in addition to the cross-validation of methylation sites identified in previous studies (11,29–33,38). To provide a robust technical analysis of RNA BS-seq data, Unique Molecular Identifiers (UMI) were introduced to estimate the error rates resulting from PCR and sequencing steps (39), and a stringent analytical pipeline was implemented to assess key parameters in m^5^C calling.

## MATERIALS AND METHODS

### Mouse neural stem cell isolation and culture

Adult mouse neural stem cells (NSCs) were isolated from the subventricular zone (SVZ) of the lateral ventricles as described previously (40). NSCs were seeded on poly-Ornithine and laminin-coated plates and cultured in DMEM/F12 medium supplemented with 2% B27 supplement, 2 mmol/l l-glutamine, 1× penicillin–streptomycin, 20 ng/ml epidermal growth factor (EGF, PeproTech), 20 ng/ml basic fibroblast growth factor (bFGF, PeproTech).

### Mitochondrial BS-seq library construction

Mitochondria were isolated from NSCs using a mitochondrial isolation kit (Abcam, ab110171) following the manufacturer's instructions. RNA was extracted from the isolated mitochondria and subjected to DNase digestion. One round of poly(A) selection was performed to enrich mitochondrial molecules. ERCC RNA mixes (Thermo) and unmethylated Xef mRNA were spiked into the samples as external RNA controls. The mitochondrial BS-seq libraries were constructed using the NEBNext® Ultra™ II Directional RNA Library Prep Kit for Illumina (NEB, E7760S) and bisulfite treatment was performed using the EZ RNA methylation kit (Zymo Research) under four different conditions: (A) bisulfite conversion using three cycles of 70°C for 10 min and 64°C for 45 min. After bisulfite conversion, RNA fragmentation and priming was performed by incubation at 94°C for 8 min in first strand reaction buffer and 6 bp random primers for first strand cDNA synthesis; (B) RNA fragmentation was performed before bisulfite conversion by incubation at 90°C for 50 s in 1× RNA fragmentation buffer and quenched by adding 1× stop buffer, then purified by Zymo Research RNA clean and concentrator-5 kit. Then, bisulfite conversion was performed using three cycles of 70°C for 10 min and 64°C for 45 min. After bisulfite conversion, RNA fragmentation was omitted and priming was performed by incubation at 65°C for 5 min in first strand reaction buffer and random primers for first strand cDNA synthesis; (C) RNA fragmentation was performed first, then bisulfite conversion was performed using three cycles of 95°C for 1 min, 70°C for 10 min and 64°C for 45 min. After bisulfite conversion, RNA fragmentation was omitted and priming was performed by incubation at 65°C for 5 min in first strand reaction buffer and random primers for first strand cDNA synthesis; (D) RNA fragmentation was performed first, then bisulfite conversion was performed using three cycles of 95°C for 1 min, 70°C for 10 min, and 64°C for 45 min. After bisulfite conversion, RNA fragmentation was omitted, and priming was performed by incubation at 65°C for 5 min in first strand reaction buffer and 6 bp ACT random hexamer primers for first strand cDNA synthesis.

### Methylation calling and post-call filtering of BS-seq reads

Raw reads were processed using fastp v0.20 (26) using the parameters (-Q -l 50 –trim_poly_x –poly_x_min_len 10). We then removed low-quality reads and trimmed read ends using the parameters (-q 25 -5 -3 -M 25 -f 6 -t 6). Clean reads were then mapped to the mm10 genome using meRanGh of the meRanTk package (25). Methylation calling was performed using meRanCall. A p-m^5^C site was defined as any C→T variants (or G→A variants in the complementary strand) compared to the converted reference genome. All p-m^5^C sites with quality above Q30 and at least 10x (C + T) coverage were called using the parameters (-mBQ 30 -sc 10 -cr 1 -mr 0.00001 -mcov 10). To achieve high-confidence in methylation calling, a ‘standard filter’ was applied to each site: (i) at least three variants ($i$) to be called at a position; (ii) the (C + T) coverage ($j$) to be 20 or greater; (iii) the methylation level, defined as $i/j$, to be at least 0.1. Bisulfite converted reads with multiple cytosines identified were considered as incomplete conversion artifacts (15,20,24). To determine the threshold of cytosines (*C*-cutoff) identified in a read, we calculated the Gini coefficient following the previously described procedure (22). After *C*-cutoff filtering, the p-m^5^C sites with ‘signal/noise’ ratios >0.9 (20,22) and FDR adjusted *P*-values less than 0.05 (14,25) were retained. Lastly, RNAfold of the ViennaRNA v2.2.9 software (–maxBPspan 150, -T 70, –MEA 0.1) was used to predict conversion-resistant regions (41). p-m^5^C sites located in these regions were removed. To ensure high-confidence in methylation calling among biological replicates, a methylated site must pass all the filtering steps described above in at least one sample and was present in at least one other replicate after the *C*-cutoff. Sites were annotated using a custom script and the Ensembl mm10 v79 GTF.

### UMI deduplication and analysis

UMIs of mitochondrial libraries were grouped and deduplicated using umi-tools (42)). Concordance and discordance rates of ERCC sequences were analyzed using a custom python script. A UMI-group was considered discordant if reads reported different nucleotides at a given variant position.

### RNA-seq library analysis

RNA-seq libraries were filtered using the same parameters applied to BS-seq libraries and mapped to the reference genome using meRanGh. The expression values for each gene were calculated using featureCounts of the Subread package suite v2.0.0 using default parameters.

### Statistical Analysis

Statistical analyses were performed using SciPy v1.7 and R v4.1.1. Fisher Exact test was used to determine differentially methylated sites among mitochondrial replicates. Wilcoxon rank-sum was used to compare methylation levels of shared m^5^C sites among RNA BS-seq libraries.

## RESULTS

### Experimental design and construction of RNA bisulfite sequencing libraries

**Figure 1.**
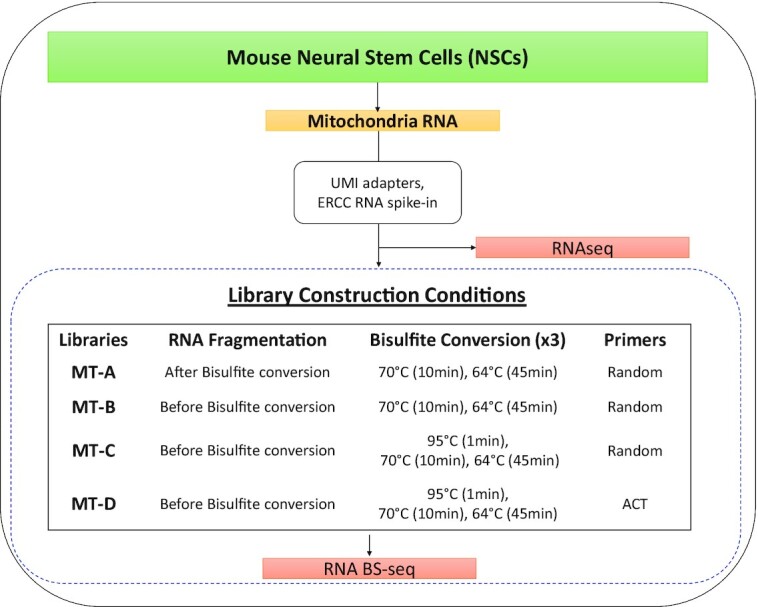
RNA library constructed in this study. Fragmentation timing, bisulfite conversion conditions, and primers used in RNA BS-seq libraries are described as individual conditions. ‘ACT’ denotes the use of ACT primers rather than random primers.

Considering the RNA bisulfite treatment conditions used in previous studies, we isolated mitochondria from mouse NSC culture and constructed RNA BS-seq libraries with four different conditions (Figure 1) to examine the impacts of: (i) the order of RNA fragmentation and bisulfite treatment; (ii) the inclusion of a heat denaturation step during bisulfite treatment and (iii) the use of random hexamers containing all four nucleotides *vs* ACT-only primers for first strand cDNA synthesis. NSCs were chosen in this study since previous reports identified RNA m^5^C methylation plays a critical role in stem cell differentiation (24,43). Western blot and RT-qPCR were performed to confirm the successful enrichment of mitochondrial isolation (Supplementary Figure S1A and B). For each condition, RNA-seq and RNA BS-seq libraries were constructed for two biological replicates and sequenced on the HiSeq 4000 platform in 150 bp paired end mode. RNA BS-seq libraries were constructed using four different procedures, which we named MT-A/B/C/D. In these libraries, adaptors carrying UMIs were used to remove PCR duplicates and assess the errors generated during PCR amplification. External RNA Controls Consortium (ERCC) consisting of pre-formulated blends of 92 transcripts were spiked in as unmethylated controls to estimate the bisulfite conversion rate. In addition to the eight RNA BS-seq libraries constructed in this study, we included an external RNA BS-seq dataset, Huang libraries, generated from mouse muscle tissues (22). Throughout this study, putative methylated sites (C in mRNA strands or G in the complementary cDNA strands) were denoted as ‘p-m^5^C’. We aimed to assess the effects of each analytical step in the pipeline for p-m^5^C identification and determine the potential sources of p-m^5^C artifacts.

### Read pre-processing and the influence of sequencing quality filter on methylation calling

Read pre-processing and cleaning are essential steps in most NGS analyses. These steps are especially critical in RNA BS-seq data processing, as sequencing artifacts heavily influence downstream analysis due to the extremely low m^5^C signal. In this study, raw reads were processed using fastp (26) to identify low-quality reads and called bases. First, non-overlapping pair-end reads and reads with lengths shorter than 50 bp after adapter trimming were discarded. For each subsequent step, this criterion was maintained. Second, reads were subjected to polyX trimming with a threshold of a 10-base nucleotide repeat. Two quality filters were applied to remove reads with: (i) an average score <Q25 and (ii) >40% of the bases with a Phred33 score less than Q25. Last, we trimmed 6 bp from the 5′ and 3′ ends of both the forward and reverse reads. This was performed to reduce the influence of methylation bias resulting from any residual bases derived from the hexamer primers used in first-strand cDNA synthesis (Supplementary Figure S2).

**Figure 2.**
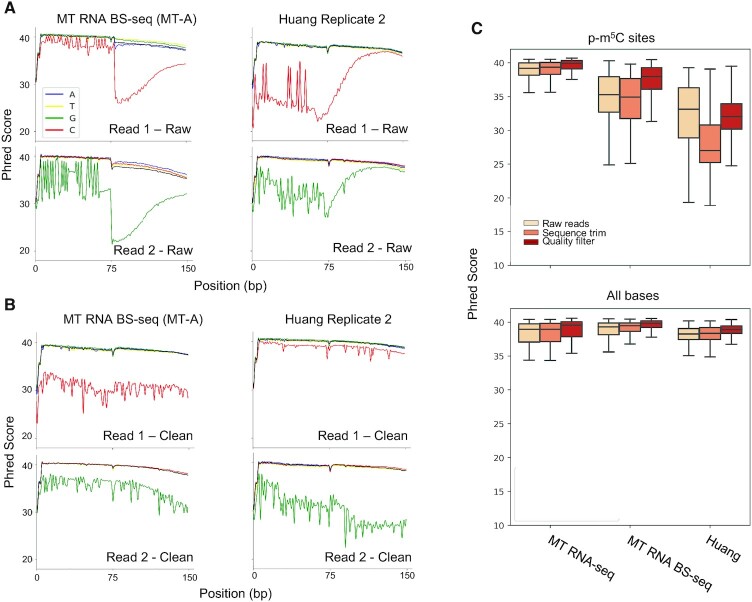
Quality score analysis of p-m^5^C bases. (A, B) Mean Phred score per base sequence of adenosine (blue), thymine (yellow), guanine (green), and cytosine (red) in MT-A replicate 1 and Huang replicate two datasets before (**A**) and after (**B**) all cleaning steps. (**C**) Mean base-level Phred scores at each step of the sequence cleaning pipeline (shades of red). p–m^5^C sites are ‘Cs’ in Read 1 and ‘Gs’ in Read 2. The p-m^5^C bases are depicted in the top figure, and the average of all nucleotides are represented in the bottom figure.

We evaluated the sequencing quality of the four types of nucleotides (A, T, C, G) at each step of read pre-processing. For libraries generated in this study, the average Phred score of cytosine in unprocessed reads was 3 points lower than those of the other three kinds of nucleotides, and 6 points lower in the RNA BS-seq dataset generated with Huang libraries (Figure 2A). The overall low sequencing quality of Cs in forward reads and Gs in reverse reads is presumably due to the composition of nucleotides in the RNA BS-seq libraries being unbalanced during sequencing. In addition, a significant drop in the Phred score of cytosine occurred starting from the 70th base position, with this trend diminishing after sequence trimming (Figure 2A and B). Such a phenomenon was observed in RNA BS-seq libraries, but not in the regular RNA-seq libraries (Supplementary Figure S3). Despite the stringent filters employed to remove low quality reads and/or bases in the pre-processing steps, the average Phred scores of p-m^5^C sites in our BS-seq libraries was 2 points lower than other nucleotides, and 7 points lower in Huang libraries. (Figure 2C). In Huang libraries, the quality of p-m^5^C sites in clean reads was one point lower on average than in raw reads due to removal of high-quality p-m^5^Cs within adaptors. Therefore, additional removal of those p-m^5^C sites with low quality scores is necessary to minimize false-positive methylation calls resulting from sequencing errors. For this reason, we included an additional Q30 cutoff filter for all p-m^5^C sites.

### Estimation of the influence of bisulfite conversion rate and PCR error on methylation calling

Using the built-in mapper functions of the meRanTk toolkit (25), all clean reads from both the RNA BS-seq and RNA-seq libraries were mapped to the mm10 reference genome (meRanGh) and transcriptome (meRanT). Sequence reads derived from RNA-seq show a higher percentage of uniquely mapped reads compared to those derived from RNA BS-seq. On average, the percentages of uniquely mapped reads using meRanGh are 52.4% and 44.4% higher than those using meRanT for RNA BS-seq and RNA-seq, respectively. We also examined the mapping efficiencies of the aggregated approach to recover multi-mapped and unmapped reads using meRanGh or meRanT alone. meRanGh alone was able to provide unique mapping rates similar to the combination of meRanGh and meRanT (Supplementary Figure S4A). More than 50% of mapped reads were mapped to exonic regions in all analyzed samples (Supplementary Figure S4B).

Using UMI adaptors and the mapping coordinates, uniquely mapped reads in this study's libraries were grouped using the ‘group’ command of the umi-tools package (42). Reads that were mapped to the same genomic coordinate and contained an identical UMI-ID were considered to be PCR amplicons. These PCR amplicons may contain small sequence variations due to PCR error, and so the most prevalent sequence was retained for methylation calling. In this study, all bisulfite converted libraries were subjected to PCR amplification to obtain enough DNA suitable for Illumina sequencing. We found that the cDNA yields of the MT-B/C/D libraries were much lower than that of the MT-A libraries. Thus, 20 cycles of PCR were performed to amplify MT-B/C/D libraries while only 16 cycles were needed for MT-A libraries. Such a difference in the number of PCR cycling across libraries was manifested by UMI-based PCR deduplication. More specifically, less than 20.0% of uniquely mapped reads in MT-A libraries were derived from PCR amplicons. Compared to those of MT-A libraries, PCR duplication rates for MT-B/C/D libraries increased by an average of 57.9% (Supplementary Table S2). This indicated that in all four conditions tested for RNA BS-seq library construction, RNA fragmentation after bisulfite sequencing (MT-A libraries) is the best in terms of cDNA yield and reducing the need for additional PCR cycles.

**Figure 3.**
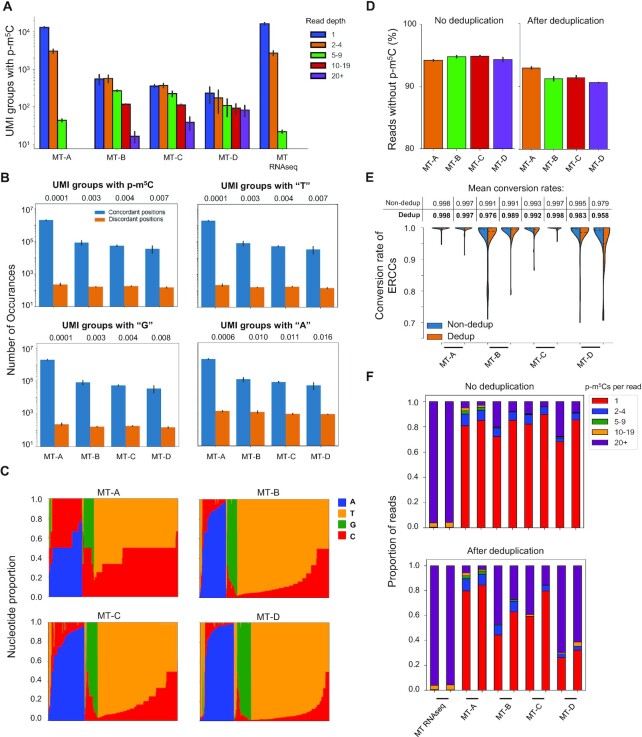
PCR deduplication and bisulfite conversion of ERCC spike-in transcripts. (**A**) UMI-duplicate coverage of ERCC transcripts containing p-m^5^C artifacts. UMI-duplicates are defined as reads containing identical UMI barcodes and mapping coordinates. (**B**) Discordance ratio of each nucleotide (p-m^5^C, T, G, A) among duplicated ERCC-mapped reads as defined above. Discordance rates were measured as the proportion of UMI-groups containing discordant positions over total UMI-groups. Read-positions with the same UMI barcode were considered concordant if the read-position shared complete similarity with all other read-positions in its group. The number of discordant reads for each nucleotide (A, T, C and G) were calculated if any dissimilarity at a position was observed. The concordance-discordance ratio is displayed above the chart. (**C**) Nucleotide frequencies of discordant positions containing p-m^5^C bases within ERCC-mapped reads. (**D**) Percent of reads without any p-m^5^C sites in ERCC reads before and after UMI-deduplication. (**E**) p-m^5^C content of ERCC-mapped reads before and after UMI-deduplication. RNA-seq and RNA BS-seq libraries are shown. p-m^5^C content was quantified and binned accordingly. (**F**) Conversion rates of individual ERCC IDs before and after UMI-deduplication. Dashed lines represent the mean conversion rate of the library. Mean conversion rates are displayed above each violin plot.

Besides PCR deduplication, the UMI-IDs also allowed for the examination of PCR errors within a UMI-group. We focused on reads mapped to ERCC references to determine PCR or sequencing error, which was reported as the discordance rate at each nucleotide position within a UMI group. As mentioned, MT-B/C/D libraries exhibited higher percentages of PCR amplicons than those of MT-A libraries. Consequently, the read depths of UMI groups identified in MT-B/C/D libraries were found to be much larger than those of MT-A libraries (Figure 3A). The increased read depth within a UMI group led to a higher probability of a PCR and/or sequencing error. Indeed, compared with MT-A libraries, discordance ratios were found to be higher in MT-B/C/D libraries (Figure 3B). Interestingly, discordance ratios were similar for three types of nucleotides (cytosine, guanine, and thymine), but the discordance ratios of adenine were two to six times higher. This is likely due to the high proportion of adenine in mRNA molecules, i.e. shorter poly-A tails not removed by the polyX filter. Importantly, for libraries generated under all four conditions, the discordant rates of p-m^5^C sites ranged from 0.1% for MT-A libraries to 0.7% for MT-D libraries. This indicates that PCR and sequencing errors at p-m^5^C sites are very low, even with 20 cycles of PCR amplification in the RNA BS-seq procedure. We further examined the nucleotide ratios at each discordant p-m^5^C site and found that C/T was the discordance type most frequently observed (Figure 3C). In addition, MT-A libraries had the highest C/T ratio while MT-D libraries had the lowest C/T ratio. This suggests an increase in PCR amplicons enriched for reads carrying thymine but not cytosine.

Since ERCC references were unmethylated spike-in controls, they were ideal for the estimation of bisulfite conversion rate. In other words, any p-m^5^C site in reads mapped to ERCC should be an artifact. Over 90% of ERCC reads in all libraries were found to be free of p-m^5^C sites. After PCR deduplication, the proportion of reads without m^5^C artifacts decreased by 1% for MT-A libraries, but 3% to 6% for MT-B/C/D libraries (Figure 3D). As a result, ERCC conversion rates for libraries MT-B/C/D decreased after deduplication (Figure 3E). Further examination of the ERCC reads carrying methylation artifacts revealed that the majority of these reads only carried one cytosine while some ERCC reads contained more than twenty cytosines. This suggests that, for some RNA molecules such as ERCC 00002/00096/000130 (Supplementary Figure S5A), bisulfite conversion reactions may not take place properly due to RNA secondary structure (13,44). In addition, PCR deduplication increased the percentages of reads carrying more than twenty cytosines, particularly for MT-B/C/D libraries (Figure 3F). This result is consistent with the observation that PCR amplification favors reads with fewer cytosines (Figure 3D). Regardless of PCR deduplication, bisulfite conversion rates for two MT-A libraries were higher than 99.7%. However, incomplete bisulfite conversion was observed in the MT-B and MT-D libraries.

Since each ERCC reference was provided with a known concentration, we further examined the influence of the bisulfite sequencing procedure on the abundance of transcripts. For the regular RNA-seq libraries, the read coverages of the ninety-two ERCC references were highly correlated with the concentrations provided by the manufacturer. Similar trends were observed in RNA BS-seq libraries except for two ERCC molecules: ERCC-00004 (7500 attomoles/ul) and ERCC-00096 (15 000 attomoles/ul). The read coverages of these two ERCCs were significantly below the expected concentrations in all mitochondrial BS-seq libraries (Supplementary Figure S5B).

To determine the transcriptome-wide effect of the bisulfite sequencing procedure, the expression levels of all mapped transcripts were determined using featureCounts (45). For RNA BS-seq libraries, the CPM (counts per million) values were determined with and without UMI-deduplication. After deduplication, the MT-B/C/D libraries reported at least a log two-fold reduction in CPM for 10.2–12.7% of genes, while <1% of genes experienced a change in expression level in MT-A libraries (Supplementary Figure S6A). We further examined the effect of bisulfite conversion on gene expression values by comparing CPM values of bisulfite converted libraries to non-converted RNA-seq libraries. MT-A RNA BS-seq libraries reported the highest correlation to the RNA-seq control with a Spearman correlation of 0.99, and only 2.6% of transcripts with changes greater than two-fold. In contrast, MT-B/C/D libraries reported over 60% of transcripts with a greater than log two-fold change (Supplementary Figure S6B). This result suggests that for the majority of genes, expression profiles remain comparable to regular RNA-seq if bisulfite sequencing libraries are constructed using the MT-A condition with 16 cycles of PCR amplification.

### Multi-level filter for highly confident methylation callings

**Figure 4.**
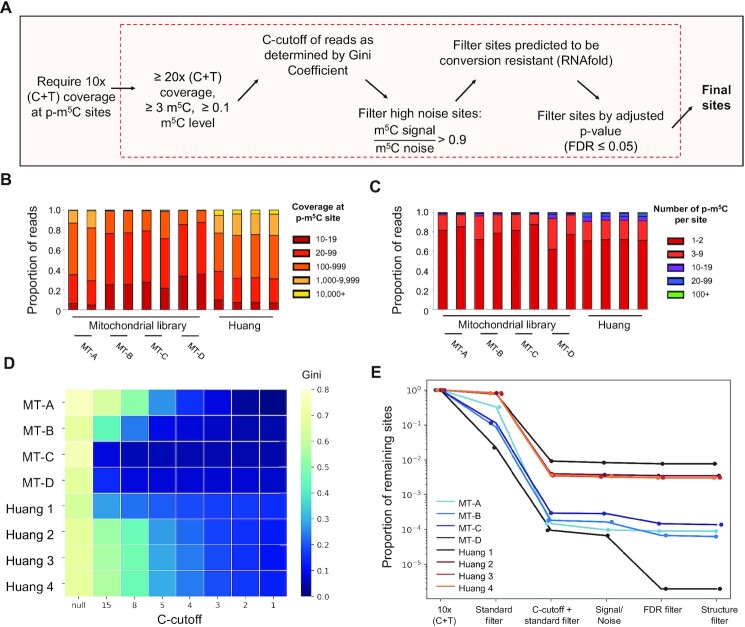
Effects of m^5^C filtering steps on bisulfite sequencing data analysis. (**A**) The summarized m^5^C filtering pipeline after methylation calling. Methylation sites were called using meRanCall. (**B**) Binned (C + T) coverage values of m^5^C sites in each bisulfite sequencing library. RNA BS-seq library replicates were merged into single libraries for m^5^C calling purposes (**C**) Binned p-m^5^C coverage per site identified. (**D**) Gini coefficient was measured after iterative C-cutoffs were performed for each library. A *C*-cutoff describes the criteria for retaining reads with multiple p-m^5^C sites. The Gini coefficients of all bisulfite-converted libraries are displayed as a heatmap. (**E**) The proportion of remaining p-m^5^C sites after each step of p-m^5^C filtering for bisulfite sequencing libraries. The original reported sites are determined using meRanCall with a 10× (C + T) coverage filter. Blue lines represent RNA BS-seq libraries generated in this study, red-orange lines represent the Huang datasets used.

After determination of p-m^5^C sites in uniquely mapped reads, multi-level filters with various strategies were widely used to achieve highly confident methylation callings (Supplementary Table S1). For each library, p-m^5^C sites with at least 10X read coverage were compiled as a starting set. We followed a multi-step filtering procedure (Figure 4A) to evaluate the influence of each filtering step on the number of methylation calling (Supplementary Table S3). The first step was a ‘Standard filter,’ which filtered sites based on the read depth and the frequencies of p-m^5^C observed for a given p-m^5^C site. Approximately 30–40% of the p-m^5^C sites identified in the MT-B/C/D libraries exhibited shallow read depths of less than 20, which may have been due to the loss of coverage from deduplication. In contrast, over 95% of p-m^5^C sites in MT-A libraries exhibited read depths over 20 (Figure 4B). The majority of p-m^5^C sites, ranging from 62%-85% across libraries, were filtered when the frequencies of p-m^5^C observed was less than three at a given site (Figure 4C).

To determine an appropriate *C*-cutoff for each library, the Gini coefficient was employed to assess the distribution of incomplete bisulfite conversion events. The C-cutoff is the threshold of cytosine identified in a bisulfite sequencing read that is considered as an incomplete conversion artifact. The Gini coefficient was calculated with the number of sites per gene (Supplementary Figure S7A) and the number of unique genes (Supplementary Figure S7B) for each sample. The Gini coefficient decreases when the p-m^5^C sites are evenly distributed across genes. By increasing C-cutoff stringency, reads which bear the largest proportion of Cs are removed resulting in a smaller Gini coefficient (Supplementary Figure S7C). For all RNA BS-seq libraries, the majority of reads carrying any candidate methylated base only had one p-m^5^C site identified (Supplementary Figure S8). Following a Gini coefficient threshold of 0.15, recommended previously (22), the C-cutoffs of MT-A and MT-B RNA BS-seq datasets were determined to be between 3 and 5. Interestingly, for MT-C/D libraries, the Gini coefficient was below 0.15 when the C-cutoff was set as 15 and 8, respectively (Figure 4D). For a given position, the frequencies of p-m^5^C observed before and after the C-cutoff were used to calculate the signal/noise ratio. p-m^5^C sites with a signal/noise ratio <0.9 were removed due to the high proportion of poorly converted reads mapped to those sites (Supplementary Table S3, Supplementary Figure S9A and B).

To delineate the filter effect on methylation calling, 100% was used as the initial number of p-m^5^C sites for each library. Combining the ‘standard filter’ with the C-cutoff filter resulted in the removal of more than 98% of p-m^5^C sites in all RNA BS-seq libraries (Figure 4E). All p-m^5^C sites identified in unmethylated ERCC reference transcripts were not able to pass the thresholds of these two filters (Supplementary Figure S10). Therefore, the combination of ‘standard filter’ with a C-cutoff filter was sufficient to minimize the chance of false-positive methylation callings. Furthermore, RNA secondary structure was predicted using the ViennaRNA package as previously reported (41) with hundreds of p-m^5^C sites found in regions predicted to be resistant to bisulfite conversion. Finally, the Benjamin-Hochberg procedure of false discovery rate (FDR) correction removed 43–95% of the remaining p-m^5^C sites in the MT-B/C/D libraries but did not remove any in libraries with bisulfite conversion rates higher than 99.9%. For datasets generated in this study, the p-m^5^C sites were retained for downstream analysis if they passed all filters in another technical replicate.

### RNA bisulfite sequencing analysis of mitochondrial mRNAs

**Figure 5.**
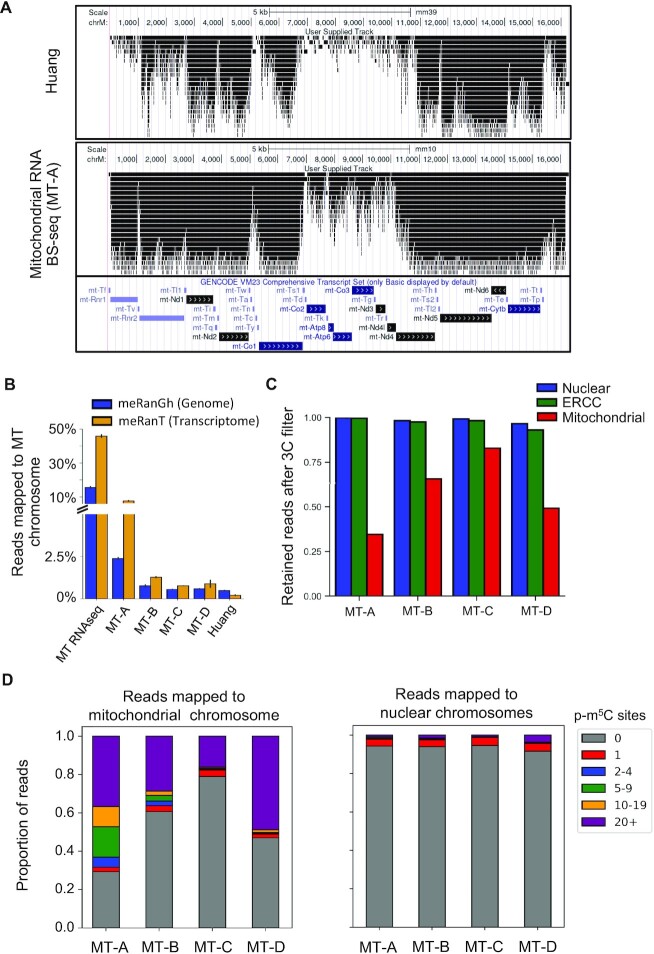
Interrogation of bisulfite preparation conditions used in mitochondrial RNA BS-seq libraries. (**A**) Read pile-up visualization of a representative Huang and Mitochondrial RNA BS-seq library MT-A using the UCSC genome browser. Peaks are scaled according to max peak height for each library. GENCODE gene annotations are displayed below. (**B**) Proportion of reads mapped to the mitochondrial chromosome using meRanGh (genome mapping) and meRanT (transcriptome mapping) for all RNA BS-seq and RNA-seq libraries. Replicates are merged, error bars represent standard deviation. (**C**) Mapped reads retained after a read *C*-cutoff of 3 for mitochondrial RNA BS-seq libraries. Non-mitochondrial (blue), ERCC (green), and mitochondrial mapped reads (red) are distinguished by color. (**D**) Number of p-m^5^C sites contained in each read, binned by p-m^5^C content. Reads are separated by mapping to the mitochondrial chromosome (left) and all other canonical chromosomes (not including control sequences) (right).

A previous study reported high methylation levels of mitochondria-related genes in heart and muscle tissues (22). The methylation of mitochondrial tRNAs and rRNAs has also been identified (11,28–33). However, the methylation of mitochondrial mRNAs remains largely unexplored. Sequencing reads mapped to the mitochondrial genome were visualized on the University of California Santa Cruz (UCSC) genome browser using Huang RNA BS-seq replicate 2 and MT-A as representatives (Figure 5C). Abundant aggregation of mapped reads centered on the coding regions of the mitochondrial chromosome were observed for both kinds of libraries. Successful enrichment of mitochondrial mRNA was demonstrated by the RNA-seq that was performed. Using the meRanT mapping tool, 45.9% of reads were mapped to the mitochondrial transcriptome and the remaining reads were mapped to nuclear transcriptomes or spike-in controls (Figure 5D). Such an enrichment for mitochondrial transcripts was more prominent in MT-A libraries than MT-B/C/D. This is likely due to performing RNA fragmentation after bisulfite conversion, resulting in a larger proportion of short RNA fragments and a greater loss of RNA template. In particularly, the median length of mt-mRNAs is much shorter than that of mRNAs derived from nuclear genome. However, despite higher proportions of mitochondrial mapped reads in MT-A, only 32.4% of those reads passed the C-cutoff filter, compared to 65.8% in MT-B and 81.1% of reads in MT-C (Figure 5E). Thus, the procedure used for bisulfite library construction can lead to a distorted proportion of mitochondrial mRNAs in the entire RNA population. Compared to regular RNA-seq libraries, the mapping rate of the mitochondrial genome was reduced approximately six to ten times in bisulfite sequencing libraries. Thus, an enrichment procedure is recommended for mitochondrial epitranscriptome studies.

Libraries constructed with enriched mitochondrial transcripts allowed us to compare epitranscriptomes derived from mitochondria and nuclear genomes. More than 95% of reads mapped to non-mitochondrial reads contained no p-m^5^C sites, while the proportion of mitochondrial mapped reads without any p-m^5^C varied from 28% (MT-A) to 79% (MT-C) (Figure 5F). While the number of p-m^5^C was low in the majority of reads mapped to the nuclear genome, a substantial portion of reads mapped to the mitochondrial genome carried >20 p-m^5^C. The non-converted reads bearing >20 p-m^5^C were found to be enriched in mitochondrial coding regions (Supplementary Figure S11). This suggests that those reads did not result from mitochondria genomic DNA contamination but rather were derived from transcripts resistant to bisulfite conversion, presumably due to intramolecular RNA secondary structure. The percentage of non-converted reads was lowest in libraries generated with the MT-C condition (Figure 5F). This suggests that RNA fragmentation after bisulfite conversion in combination with a high temperature bisulfite conversion step may be the most suitable for generation of RNA BS-seq data.

Libraries generated in this study were constructed with an equal aliquot from the same pool of RNAs, which allowed us to examine the influence of the four experimental procedures on methylation data generation. For pair-wise comparisons, we identified the p-m^5^C sites shared in libraries generated with two different conditions (Supplementary Figure S12A). The methylation level correlations were found to have a Spearman coefficient above 0.75 (Supplementary Figure S12B). We further performed differential methylation analysis and identified 7, 1, and 0 differentially methylated sites (DMSs) in the pair-wise comparisons of MT-A *vs* MT-B, MT-B versus MT-C, and MT-C *vs* MT-D, respectively. All DMSs were removed from the list of high-confidence sites. The use of random primers during 1st strand cDNA synthesis has commonly been used in RNA-bisulfite studies, while ACT primers have been suggested to avoid reverse transcription of inefficiently deaminated RNA templates (16,46). In this study, we did not observe a significant advantage of using ACT primers.

**Figure 6.**
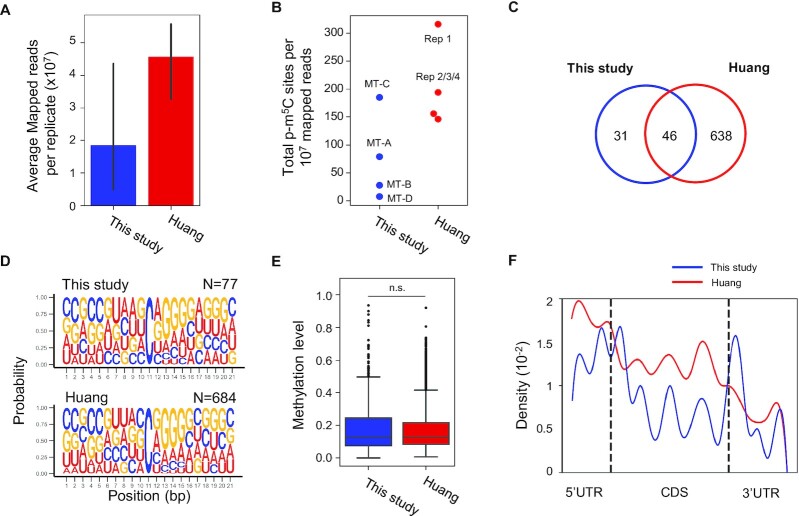
Characterization of m^5^C sites among cellular compartments of mouse NSCs. (**A**) Average mapped read count for bisulfite-converted libraries. Bars represent standard deviation. (**B**) Methylation calling efficiencies of m^5^C sites per 10^7^ mapped reads. Libraries constructed in this study and Huang replicates are shown in blue and red, respectively. (**C**) Overlap of high-confidence m^5^C sites among libraries generated in this study and Huang muscle tissue datasets. (**D**) Sequence logo surrounding the high-confidence m^5^C sites. (**E**) Methylation level of the high-confidence m^5^C sites. Significance was tested using Wilcoxon Rank-Sum. (**F**) Distribution of m^5^C sites across binned mRNA transcripts. 5′UTR and 3′ UTR positions are indicated by dashed lines at bins 5 and 18, respectively.

We further compared the methylation profiles of RNAs obtained with four different conditions (Supplementary Table S4). Highly confident m^5^C sites were defined as Ensembl-annotated p-m^5^C sites which passed all filtering criteria in at least one replicate and contained at least one m^5^C count and 10x read coverage after the C-cutoff in another replicate. Using the above criteria, 77 and 684 sites were identified to be m^5^C sites with high confidence in this study and the Huang dataset respectively (Supplementary Table S5). Library MT-C reported a m^5^C site per mapped read rate comparable to Huang and MT-A libraries despite containing ∼40 million fewer reads (83.2% fewer) (Figure 6A and B). Replicates from the Huang study reported 61.0% of high-quality sites present in at least two replicates, and 37.7% of sites were present in all four replicates (Supplementary Figure S13A and B). Of high-confidence sites identified in this study's libraries, 59.7% were also identified in Huang libraries, suggesting some m^5^C sites may be unique to NSCs (Figure 6C).

As reported previously (20,22,47), a ‘GGG’ motif was identified downstream of the m^5^C sites of high confidence (Figure 6D). Interestingly, we found that the m-bias filter was able to remove a strong 5′GGG motif upstream of p-m^5^C sites (Supplementary Figure S14), which was suggested to be an indication of false positive sites (47). The difference in methylation levels of high-confidence m^5^C sites was insignificant (Wilcoxon rank-sum, *P >* 0.05) (Figure 6E). Analysis of m^5^C distribution on mRNA transcripts was calculated as previously reported (14,20,22). Analysis revealed sites biased to the 5′UTR of mRNAs, with the lowest density in the 3′UTR (Figure 6E). Our characterization of high-confidence m^5^C sites in this study reveals features consistent with previously established reports (20,22,47), namely the down-stream ‘GGG’ motif and the enrichment near the transcription starting sites of mRNA transcripts. In mitochondria 12S ribosomal RNA (MT 911, mt-Rnr1), one heavily methylated p-m^5^C site was found to have a methylation level above 80% in all four conditions and in the published Huang dataset (22). However, we were not able to consistently identify any p-m^5^C sites with high confidence on mitochondrial mRNAs (Supplementary Table S6).

## DISCUSSION

Substantial differences in the prevalence and magnitude of mRNA methylation reported call into question whether the best practice of RNA BS-seq data generation and analysis has been achieved (22,47). In this study, we examined the impact of key parameters in both experimental and computational procedures on the detection of RNA cytosine methylation.

Using the established RNA bisulfite analysis pipeline, RNA BS-seq data was analyzed in a systematic fashion. We observed that the procedure for bisulfite library construction reduced the proportion of sequence reads mapped to the mitochondrial genome. Compared with transcripts derived from the nuclear genome, the overall bisulfite conversion rate of mitochondrial transcripts was poor. More specifically, after bisulfite conversion, a substantial percentage of mitochondrial transcripts had over twenty cytosines. This may be due to the intramolecular secondary RNA structure within mitochondrial transcripts. Although no m^5^C sites on mitochondrial mRNAs could be determined with high confidence for mouse neural stem cells, we confirmed a highly methylated cytosine on mitochondrial rRNA as previously reported (22,30). However, we enriched for poly-A selected mitochondrial transcripts which are likely to be mature or to-be-degraded mRNAs. In particularly, polyadenylated truncated mitochondrial transcripts has been associated with polyadenylation-dependent RNA degradation in human mitochondria (48). Thus, we could not rule out the possibility that some cytosines in primary mitochondrial transcripts are methylated.

Previous studies have conflicting viewpoints regarding performing RNA fragmentation before or after bisulfite conversion (16,22). We found that RNA fragmentation performed after bisulfite conversion (condition MT-A) significantly improved the yield of the cDNA library, compared with MT-B/C/D conditions. Utilizing UMIs, we observed that the PCR error rate positively correlates with the number of PCR cycles and PCR favors unmethylated templates. Such a bias in PCR amplification of sequences carrying thymidine *vs* cytosine may lead to an underestimation of the methylation level. In addition, the inclusion of a high-temperature treatment helps to reduce the proportion of unconverted reads originating from the mitochondrial genome. Altogether, our study recommends the following procedures for RNA bisulfite sequencing study: (i) perform RNA fragmentation after bisulfite conversion; (ii) include a high-temperature denaturation step in bisulfite treatment cycling and (iii) include a UMI-deduplication strategy for low-input RNA samples or amplify the library with a low number (<16) of PCR cycling.

One important characteristic of RNA BS-seq data is the low Phred scores of p-m^5^C sites. The stringent filters employed to remove low quality reads and/or bases in the pre-processing steps help but cannot fully compensate the difference in sequencing quality between the p-m^5^C sites and the other three kinds of nucleotides. A previous study indicated that an upstream ‘GGG’ motif was frequently associated with false positive sites (47). We found that the m-bias filter was able to remove sites with such a motif. In addition, all false positive p-m^5^C sites in the ERCC reference controls were removed when the *C*-cutoff filter was applied together with the ‘Standard filter’. Therefore, our study supports the following parameters/steps in methylation calling: (i) an additional quality filter with Q30 as a cutoff for all p-m^5^C sites; (ii) a stringent m-bias correction and (iii) a combination of a ‘Standard filter’ with the C-cutoff filter. In summary, our study conducted a systematic evaluation of parameters used in RNA bisulfite sequencing and may shed new light on RNA methylation data generation and analysis. Further improvement may be achieved with improved characterization of false-positive sites (47), alternative deamination techniques (49), and advance computational modeling for m^5^C calling (50).

## DATA AVAILABILITY

Data generated in this study have been submitted to the NCBI Gene Expression Omnibus under accession number GSE190614. Analyses in this study was performed using the R v4.1.1, and Python 3.9.4 packages Biopython v1.78, matplotlib v3.3.4, Seaborn v0.11, and Pysam v0.16. The software package developed in this study is available in GitHub repository (https://github.com/zaustinj33/SysAnalysisRNABS).

## Supplementary Material

lqac045_Supplemental_Files
