# Supplementary material for: Systematic evaluation of parameters in RNA bisulfite sequencing data generation and analysis
Source: NAR Genom Bioinform. 2022 Jun 3;4(2):lqac045. doi: 10.1093/nargab/lqac045 (PMC9164272; doi:10.1093/nargab/lqac045)
Supplement: lqac045_Supplemental_Files [file lqac045_supplemental_files.zip › Supplementary figures.pdf]

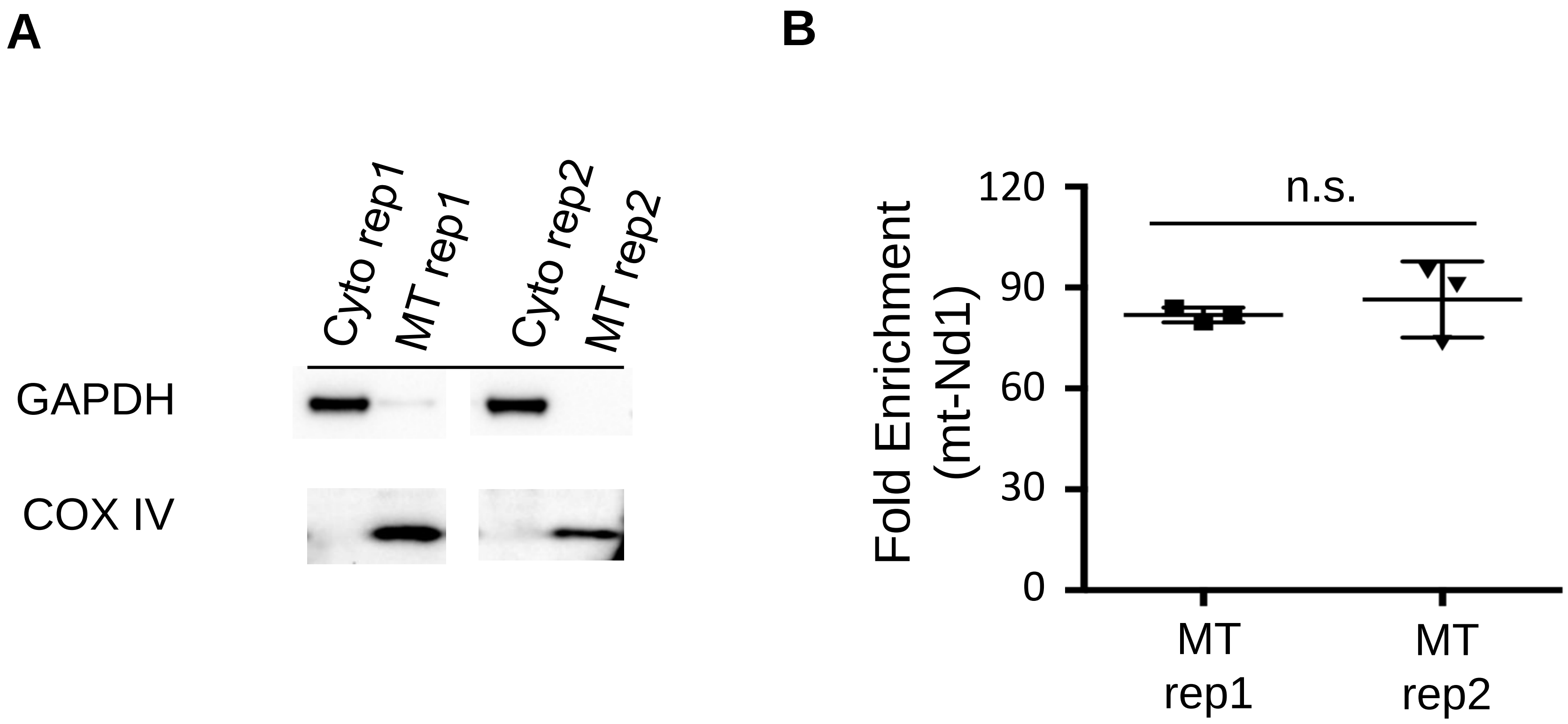

Supplementary Figure 1. (A) Western blot of mouse NSC cell fractions representing cytosol and mitochondrial structures. GAPDH was used as a marker for cytosol, while COX IV was used as a marker for the mitochondria. (B) qPCR assay of mt-Nd1 in mitochondrial fractions. Bar indicates mean and S.E.M of biological replicates.

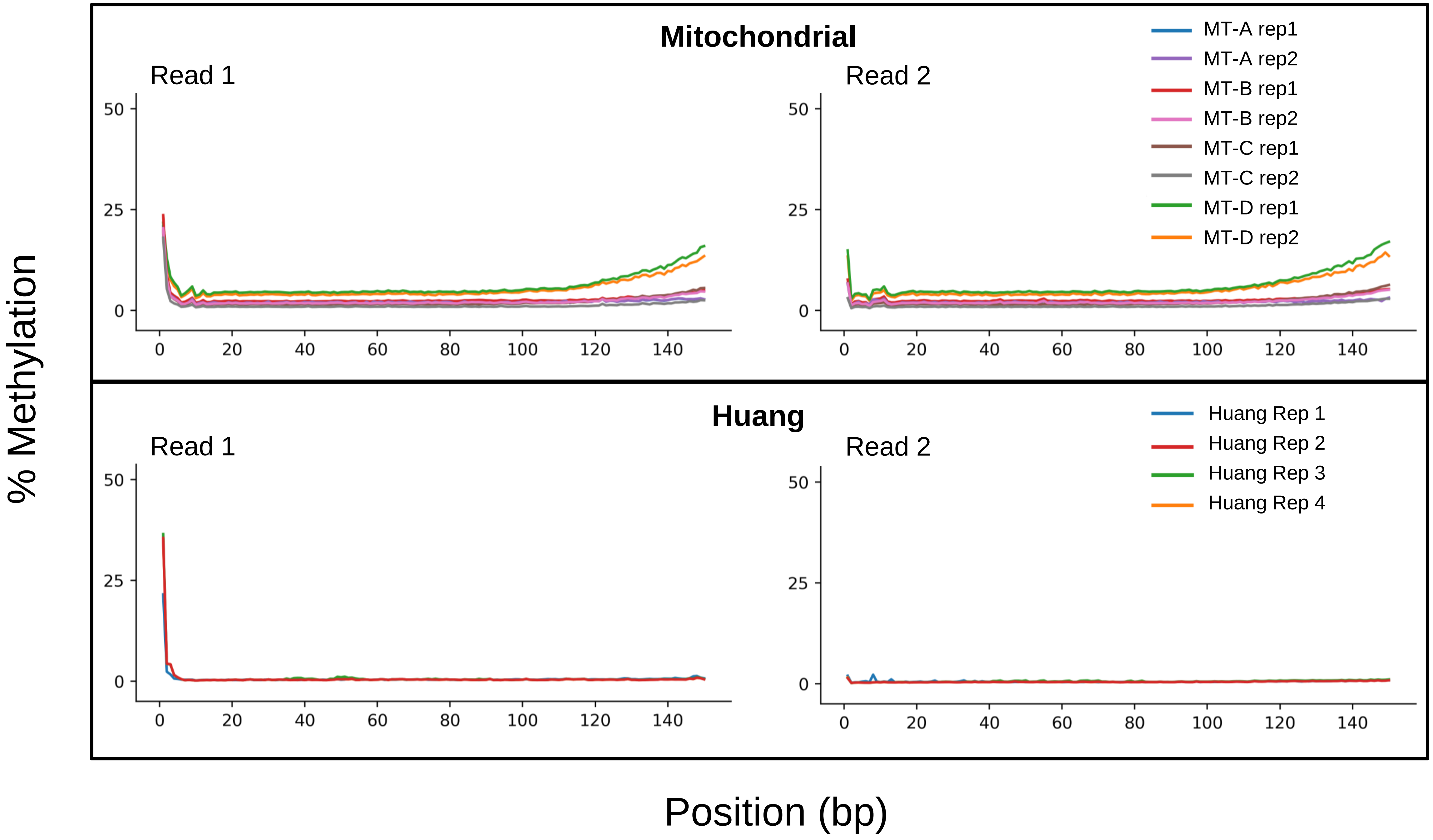

Supplementary Figure 2. Methylation bias of RNA bisulfite libraries. Methylation bias was calculated using the Bismark tool bismark_methylation_extractor [48]. Color code indicates the replicate.

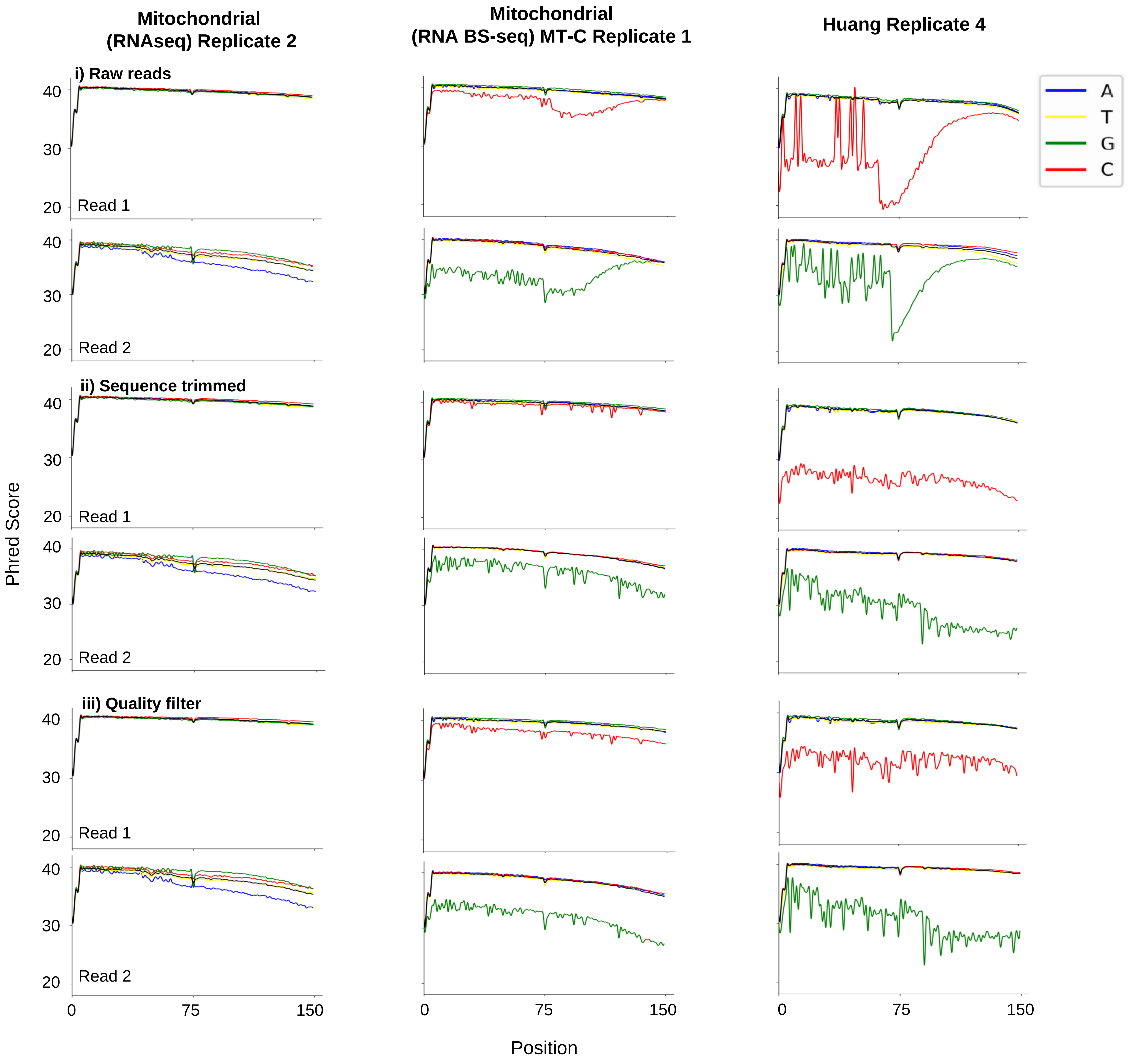

Supplementary Figure 3. Mean per base sequence quality of Adenosine (blue), Thymine (yellow), Guanine (green), and Cytosine (red). A representative sample was chosen from each RNA BS-seq and RNA-seq library, with both Read 1 and Read 2 shown.

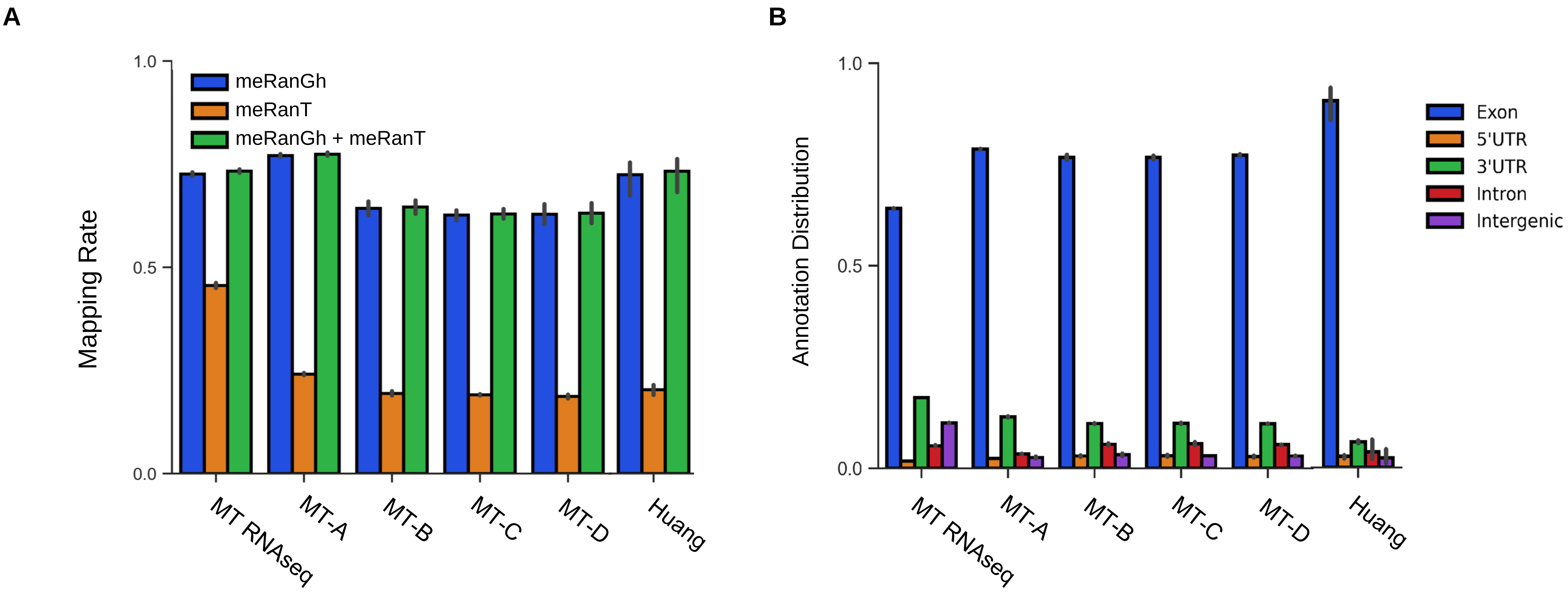

Supplementary Figure 4. **(A)** Mapping rate of libraries using meRanGh (genome) and meRanT (transcriptome). Hybrid mapped reads were created by piping multi-mapped and unmapped reads to the unused mapping protocol (i.e., meRanGh multi-mapped and unmapped reads were used as input to meRanT to create aggregate Genome-mapped reads). Only uniquely mapped reads are reported. **(B)** Mapped transcripts were annotated using RSeQC [49] and RefSeq mm10 annotations.

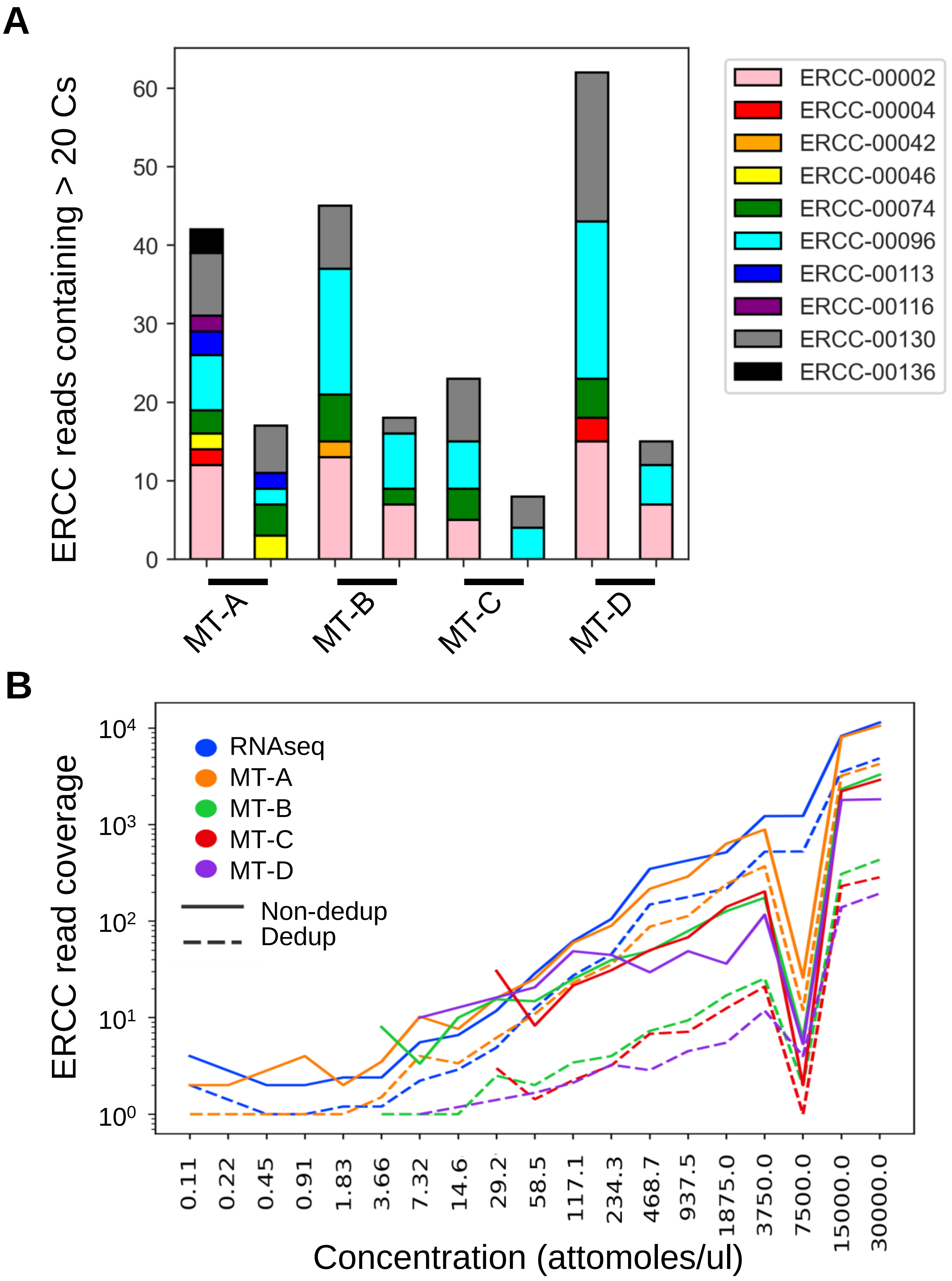

Supplementary Figure 5. **(A)** ERCC coverage of reads containing more than 20 p-m^{5}C artifacts (minimum 2 reads). **(B)** ERCC coverage compared to input ERCC concentration in non-UMI-deduplicated libraries and their corresponding deduplicated libraries. Reads were mapped using meRanGh. Concentrations were prepared according to the ThermoFisher protocol (Methods). Each concentration listed contains multiple unique ERCCs.

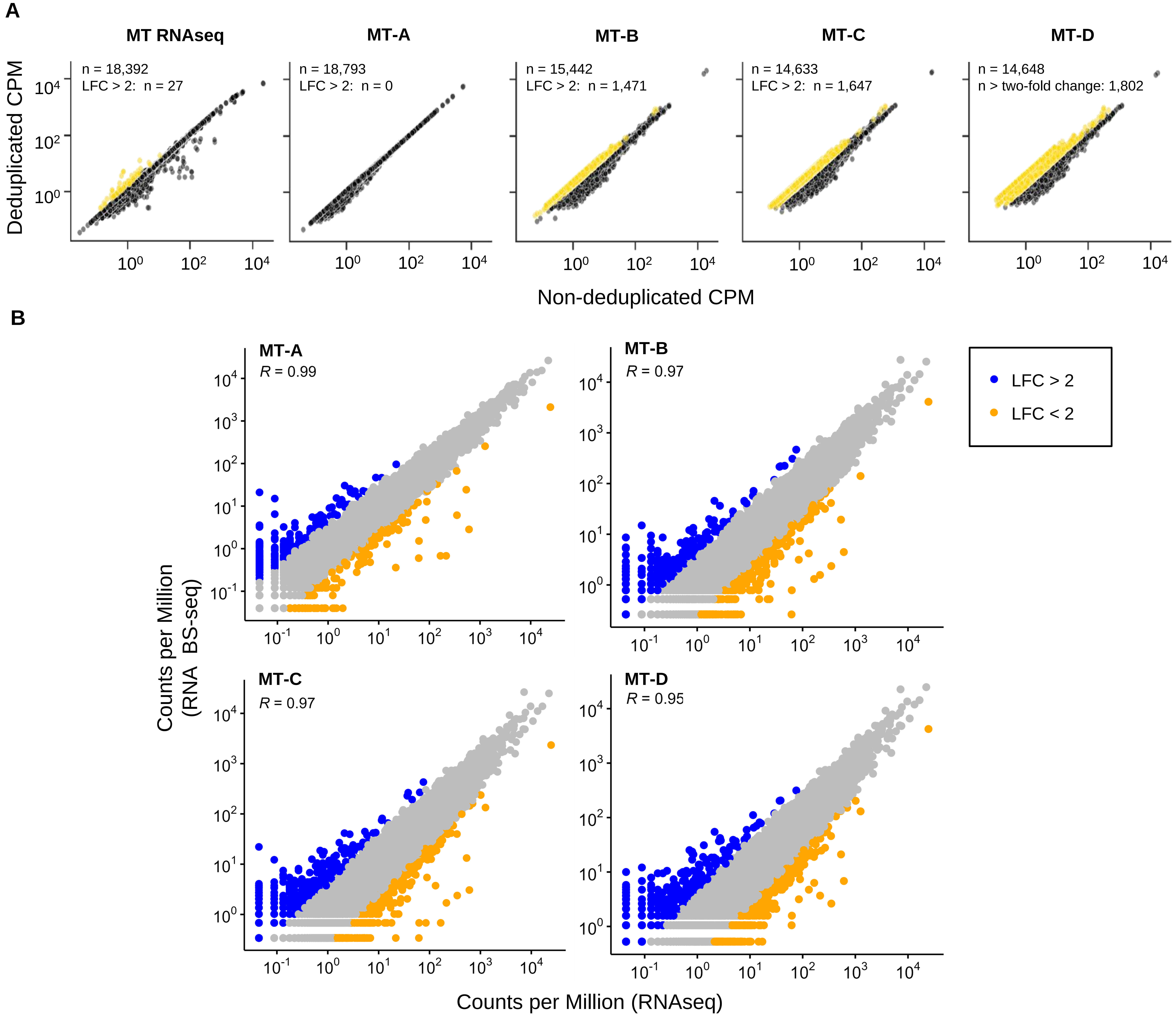

Supplementary Figure 6. **(A)** Counts per million values of deduplicated and non-deduplicated libraries were calculated from meRanGh mapped files using featureCounts. Genes that experienced more than two log-fold change after deduplication are highlighted in yellow.. **(B)** CPM values of bisulfite libraries were compared to non-converted RNA-seq libraries. Genes with higher than two log-fold change are highlighted in blue, genes with less than negative two log-fold change are highlighted in orange. All other genes are colored in grey.

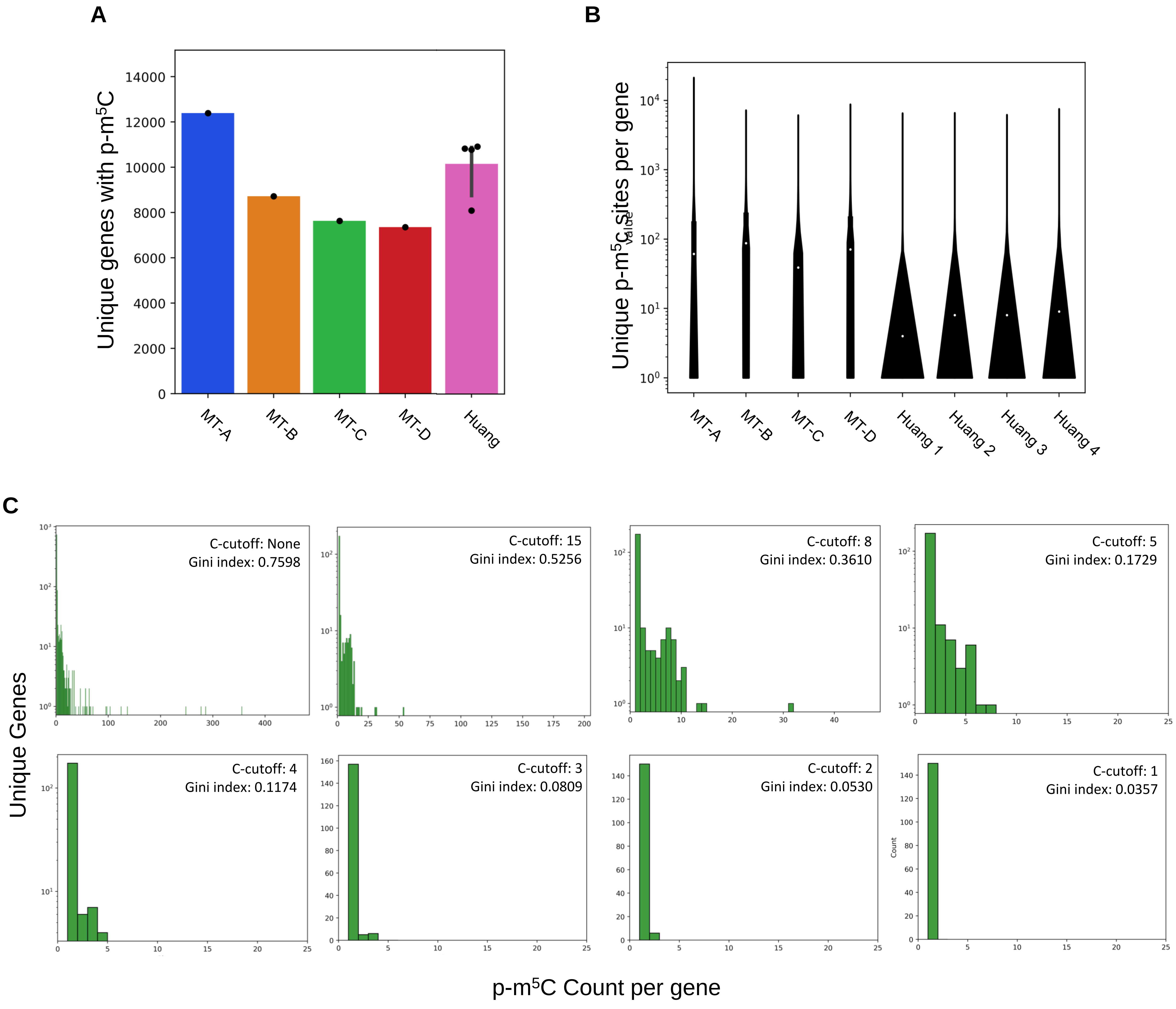

Supplementary Figure 7. **(A)** Number of unique genes that carry p-m^{5}C sites with at least 10x coverage. **(B)** Number of unique p-m^{5}C site locations with at least 10x coverage for each bisulfite converted library. **(C)** Effect of the C-cutoff filter on the number of unique genes in a library. MT-A is used as a representative sample.

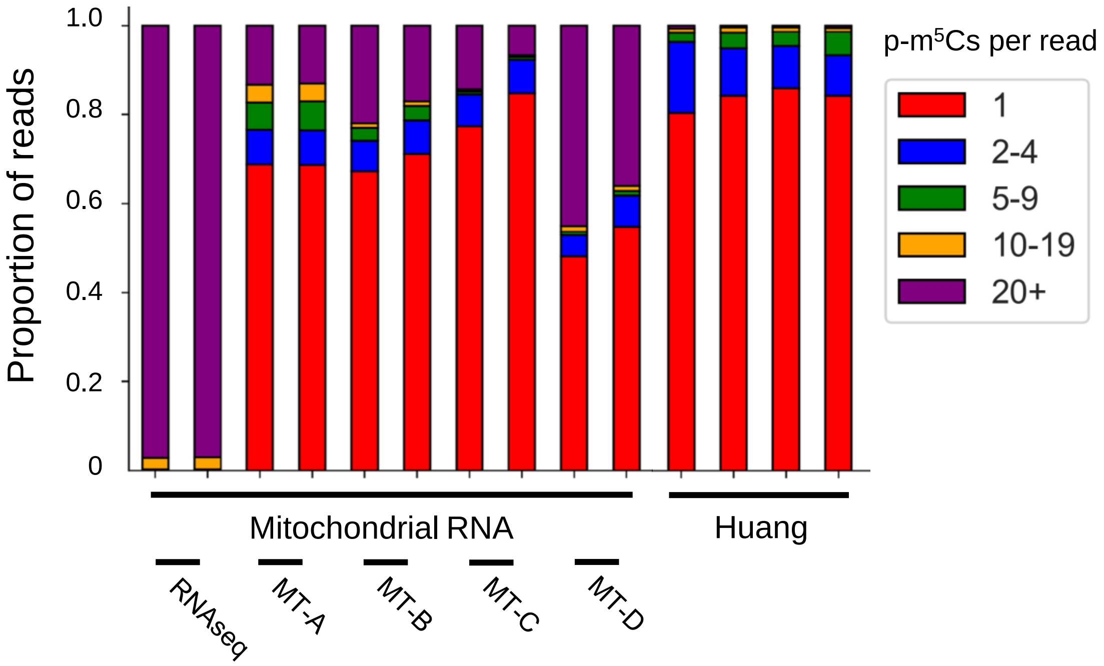

Supplementary Figure 8. Binned C counts of each library. Number of p-m^{5}C occurrences in every read was quantified, then binned accordingly. Reads with 0 p-m^{5}Cs are not shown.

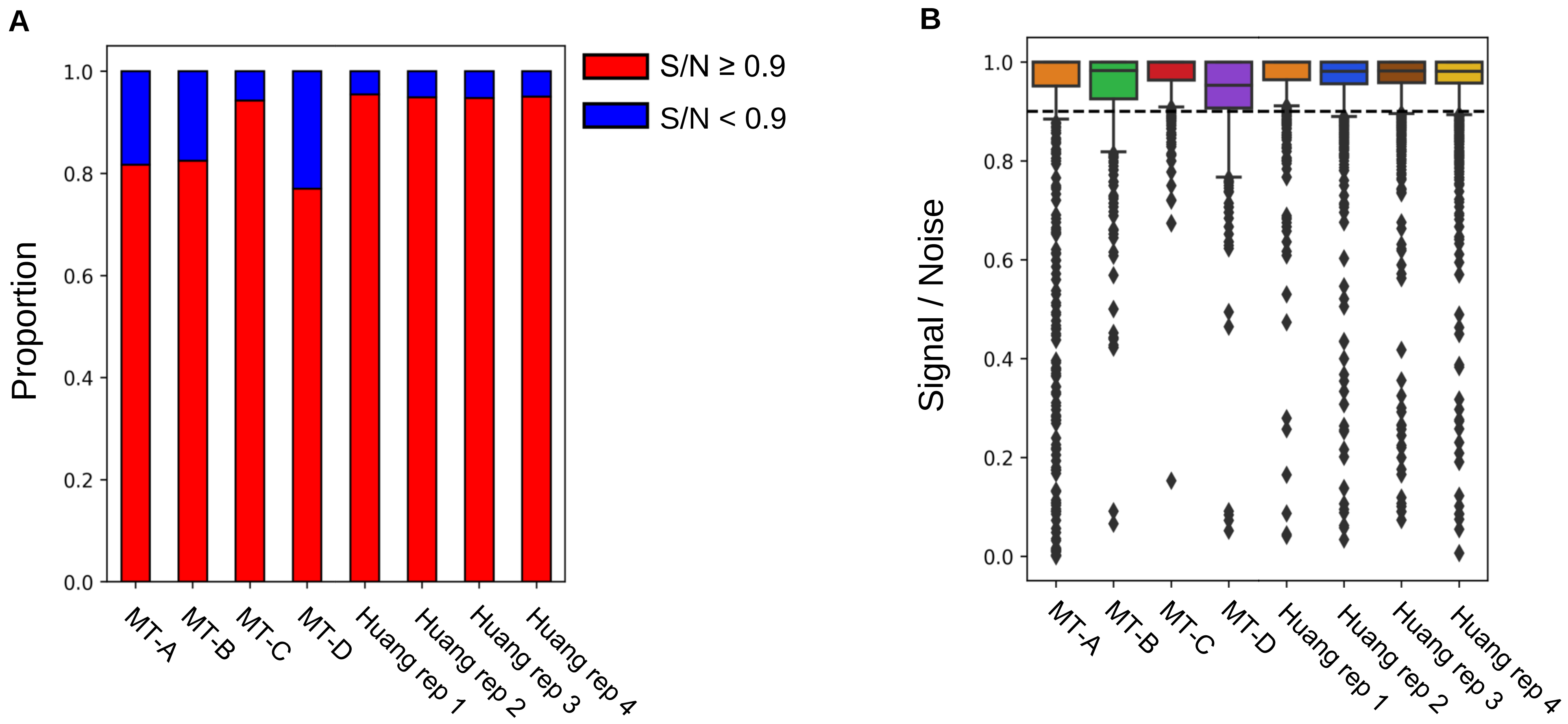

Supplementary Figure 9. **(A)** Percentage of sites above and below the signal/noise criteria **(B)** Distribution of signal/noise among all libraries. Dotted line represents the 0.9 cutoff.

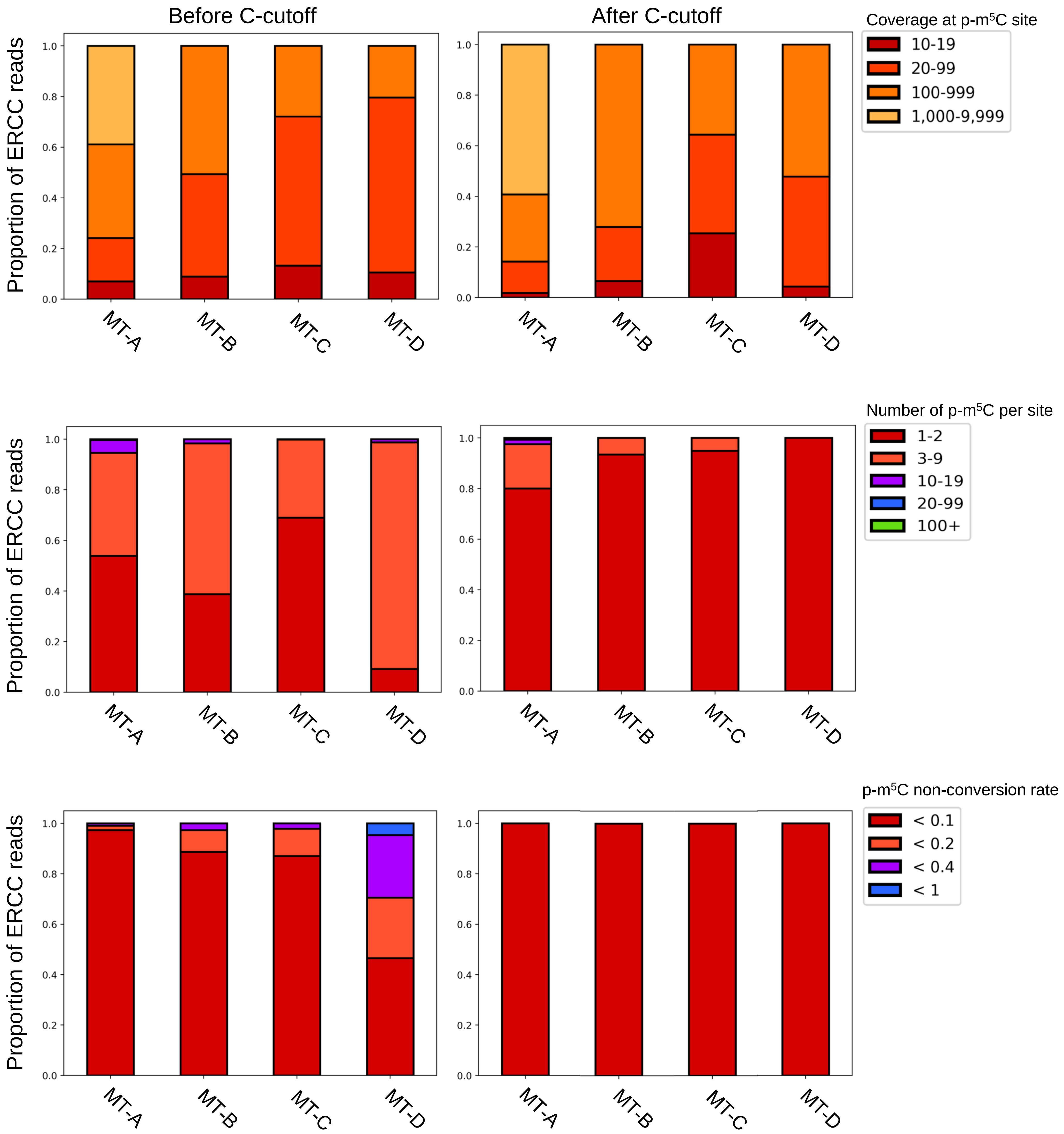

Supplementary Figure 10. Read coverage, m^{5}C artifact count, and m^{5}C artifact ratio at ERCC positions. Values are binned according to the legend.

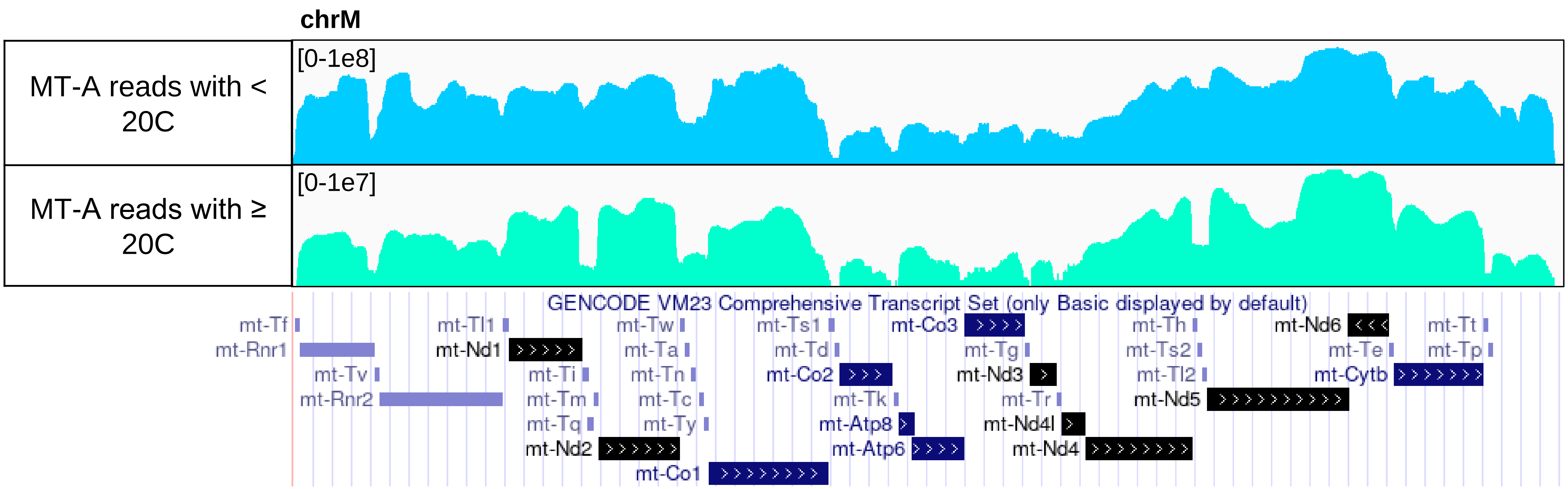

Supplementary Figure 11. Read pile-up of MT-A along the mitochondrial chromosome. Reads containing fewer than p-m^{5}C sites are displayed in cyan, while reads containing 20 or more p-m^{5}C sites are displayed in teal. GENCODE gene annotations are displayed below.

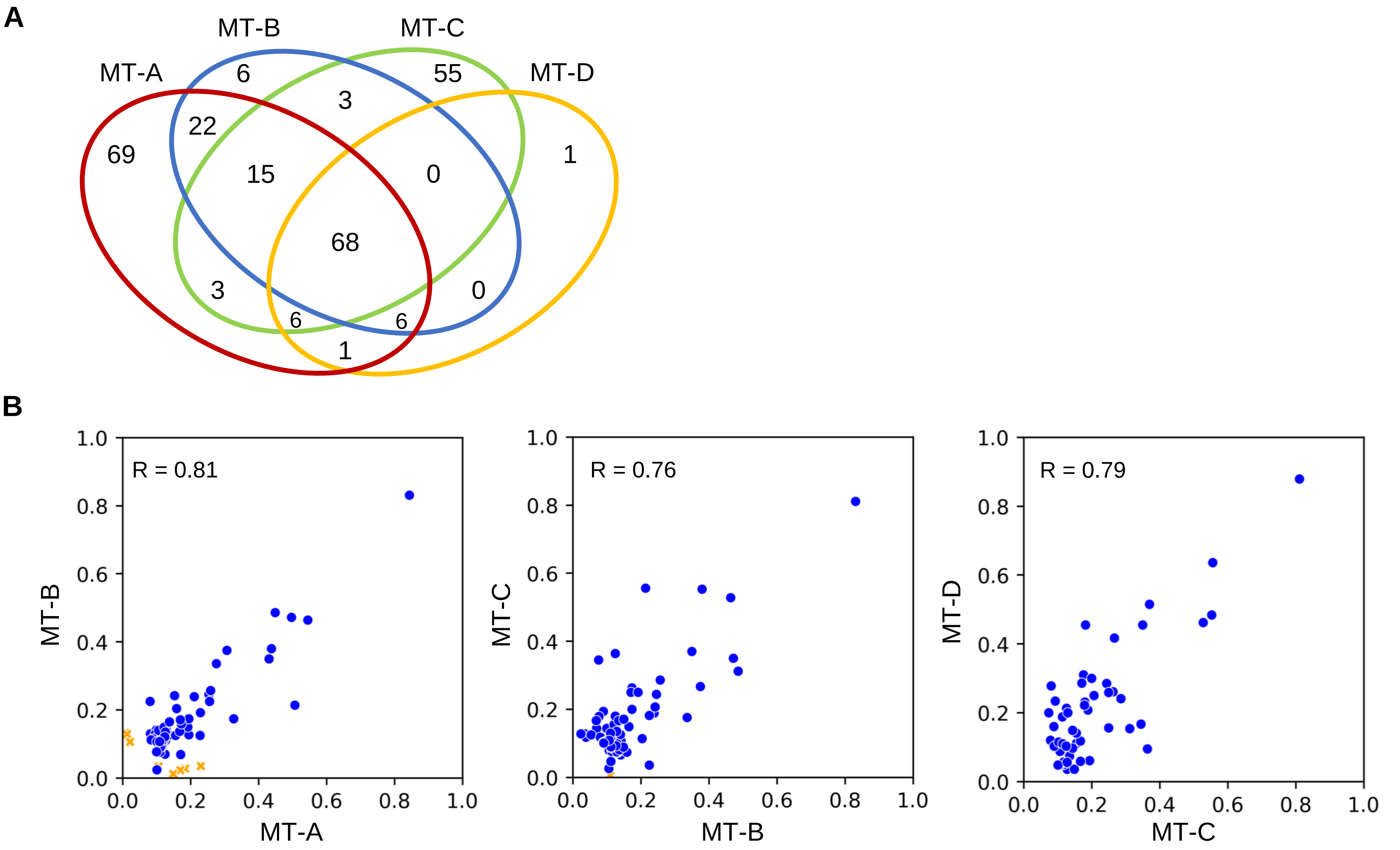

Supplementary Figure 12. **(A)** Overlap of m^{5}C sites present in mitochondrial libraries. **(B)** Correlation of methylation level in sites present in at least one condition. Differentially methylated sites are indicated by an orange "X" (Fisher Exact test, Benjamini-Hochberg adjusted *p*-value < 0.01).

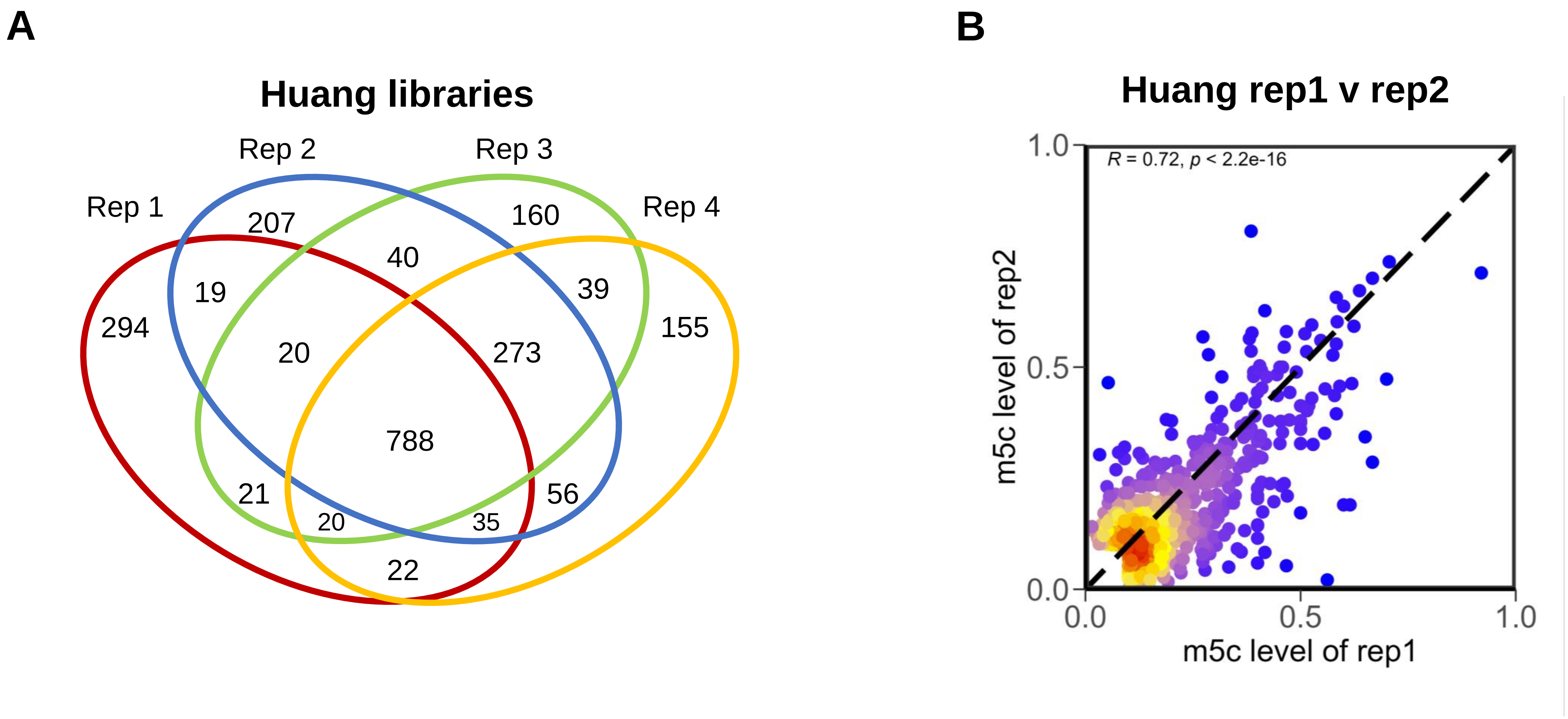

Supplementary Figure 13. **(A)** Site overlap positions of all bisulfite-converted libraries used in this study. **(B)** Correlation plots of sites present in at least one replicate. Huang replicates 1 and 2 are used as representative samples. Spearman's *R* value is shown. Points are colored according to density (red: high density, blue: low density).

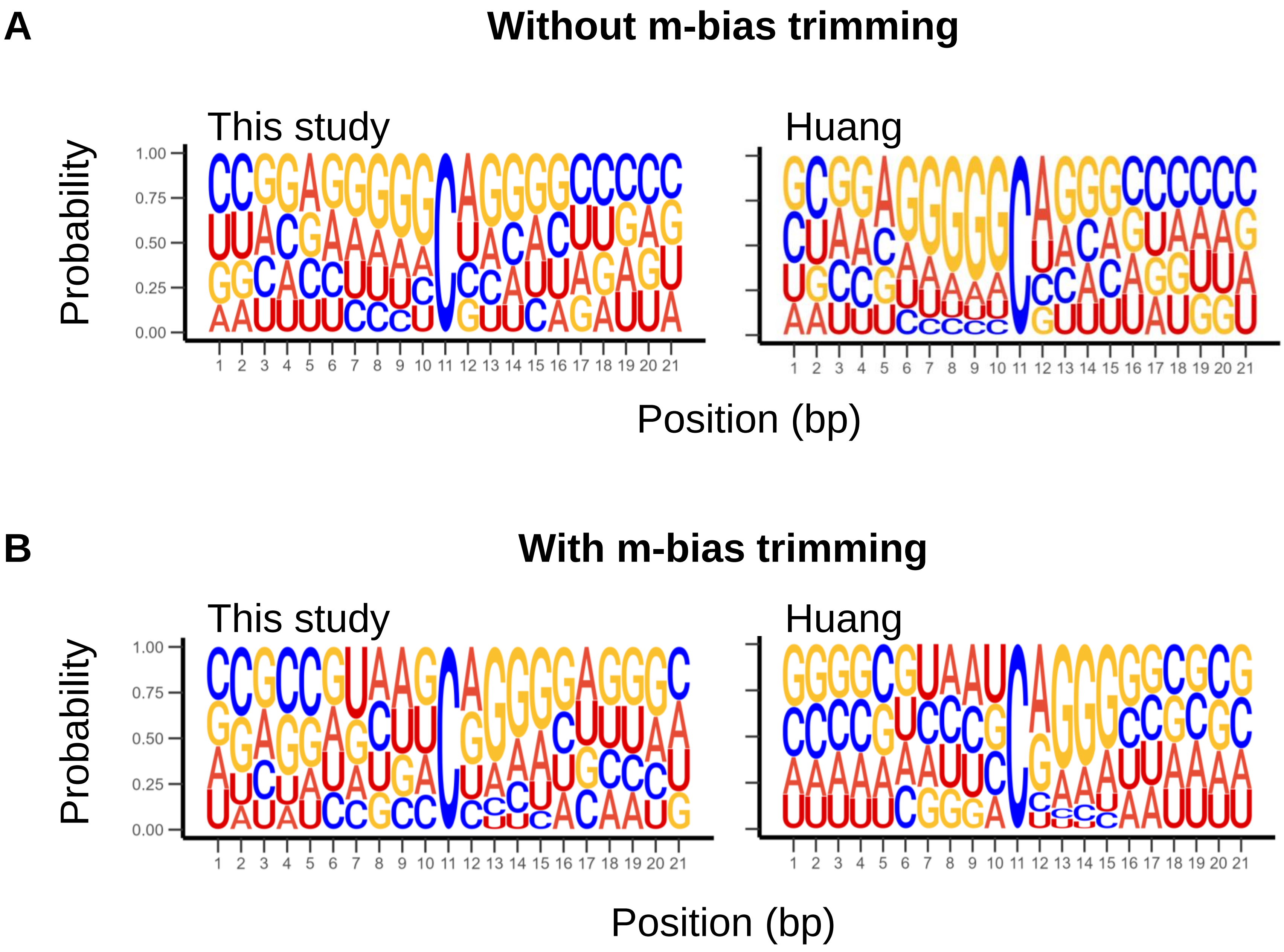

Supplementary Figure 14. Sequence logo of p-m^{5}C sites that passed all pipeline filters without **(A)** and with **(B)** 6 bp read-end trimming.
