# Supplementary material for: Systematic evaluation of parameters in RNA bisulfite sequencing data generation and analysis
Source: NAR Genom Bioinform. 2022 Jun 3;4(2):lqac045. doi: 10.1093/nargab/lqac045 (PMC9164272; doi:10.1093/nargab/lqac045)
Supplement: lqac045_Supplemental_Files [file lqac045_supplemental_files.zip › Supplementary_information.docx]

# Supplementary information

**Supplementary Table 1**. Non-exhaustive list of RNA-BS preparation and analysis methodologies.

**Supplementary Table 2**. Sequencing library pre-processing statistics.

**Supplementary Table 3**. Effects of methylation calling filtering on bisulfite libraries.

**Supplementary Table 4**. Count matrix of all m^5^C sites identified in this study.

**Supplementary Table 5**. High-confidence m^5^C sites identified in this study and Huang datasets.

**Supplementary Table 6**. Mitochondrial m^5^C sites identified in this study.

**Supplementary Figure 1. (A)** Western blot of mouse NSC cell fractions representing cytosol and mitochondrial structures. GAPDH was used as a marker for cytosol, while COX IV was used as a marker for the mitochondria. **(B)** qRT-PCR assay of mt-Nd1 in mitochondrial fractions. Bar indicates mean and S.E.M of biological replicates.

**Supplementary Figure 2**. Methylation bias of RNA bisulfite libraries. Methylation bias was calculated using the Bismark tool bismark_methylation_extractor [51].

**Supplementary Figure 3**. Mean per base sequence quality of Adenosine (blue), Thymine (yellow), Guanine (green), and Cytosine (red). A representative sample was chosen from each RNA BS-seq and RNA-seq library, with both Read 1 and Read 2 shown.

**Supplementary Figure 4**. **(A)** Mapping rate of libraries using meRanGh (genome) and meRanT (transcriptome). Hybrid mapped reads were created by piping multi-mapped and unmapped reads to the unused mapping protocol (i.e., meRanGh multi-mapped and unmapped reads were used as input to meRanT to create aggregate Genome-mapped reads). Only uniquely mapped reads are reported. **(B)** Mapped transcripts were annotated using RSeQC [52] and RefSeq mm10 annotations.

**Supplementary Figure 5**. **(A)** ERCC coverage compared to input ERCC concentration in non-UMI-deduplicated libraries and their corresponding deduplicated libraries. Reads were mapped using meRanGh. Concentrations were prepared according to the ThermoFisher protocol (Methods). Each concentration listed contains multiple unique ERCCs. **(B)** ERCC coverage of reads containing more than 20 p-m^5^C artifacts (minimum 2 reads).

**Supplementary Figure 6**. **(A)** Counts per million values of deduplicated and non-deduplicated libraries were calculated from meRanGh mapped files using featureCounts. Genes that experienced more than two log-fold change after deduplication are highlighted in yellow. **(B)** CPM values of bisulfite libraries were compared to non-converted RNA-seq libraries. Genes with higher than two log-fold change are highlighted in blue, genes with less than negative two log-fold change are highlighted in orange. All other genes are colored in grey.

**Supplementary Figure 7**. (**A)** Number of unique genes that carry p-m^5^C sites with at least 10x coverage. (**B)** Number of unique p-m^5^C site locations with at least 10x coverage for each bisulfite converted library. (**C)** Effect of the C-cutoff filter on the number of unique genes in a library. MT-A is used as a representative sample.

**Supplementary Figure 8**. Binned C counts of each library. Number of p-m^5^C occurrences in every read was quantified, then binned accordingly. Reads with 0 p-m^5^Cs are not shown.

**Supplementary Figure 9**. **(A)** Percentage of sites above and below the signal/noise criteria **(B)** Distribution of signal/noise among all libraries. Dotted line represents the 0.9 cutoff.

**Supplementary Figure 10**. Read coverage, m^5^C artifact count, and m^5^C artifact ratio at ERCC positions. Values are binned according to the legend.

**Supplementary Figure 11**. Read pile-up of MT-A along the mitochondrial chromosome. Reads containing fewer than p-m^5^C sites are displayed in cyan, while reads containing 20 or more p-m^5^C sites are displayed in teal. GENCODE gene annotations are displayed below.

**Supplementary Figure 12**. **(A)** Overlap of m^5^C sites present in mitochondrial RNA BS-seq libraries. **(B)** Correlation of methylation level in sites present in at least one condition. Differentially methylated sites are indicated by an orange “X” (Fisher Exact test, Benjamini-Hochberg adjusted *p*-value < 0.01).

**Supplementary Figure 13**. **(A)** Site overlap positions of all bisulfite-converted libraries used in this study. **(B)** Correlation plots of sites present in at least one replicate. Huang replicates 1 and 2 are used as representative samples. Spearman’s *R* value is shown. Points are colored according to density (red: high density, blue: low density).

**Supplementary Figure 14.** Sequence logo of p-m^5^C sites that passed all pipeline filters without **(A)** and with **(B)** 6 bp read-end trimming.
